# Supplementary material for: Selective Passivation of Three-Dimensional Carbon Microelectrodes by Polydopamine Electrodeposition and Local Laser Ablation
Source: Micromachines (Basel). 2022 Feb 26;13(3):371. doi: 10.3390/mi13030371 (PMC8950879; doi:10.3390/mi13030371)
Supplement: Supplementary file 1 [file micromachines-13-00371-s001.zip › micromachines-1576227-supplementary.pdf]

micromachines

Supplementary Materials: Selective Passivation of Three-Dimensional Carbon Microelectrodes by Polydopamine Electrodeposition and Local Laser Ablation

Babak Rezaei *,†, Saloua Saghir †, Jesper Yue Pan, Rasmus Schmidt Davidsen and Stephan Sylvest Keller

National Centre for Nano Fabrication and Characterization, DTU Nanolab, Technical University of Denmark, 2800 Konges Lyngby, Denmark; saghir.saloua@gmail.com (S.S.); jesyup@dtu.dk (J.Y.P.); rasda@dtu.dk (R.S.D.); suke@dtu.dk (S.S.K.)

* Correspondence: barez@dtu.dk; Tel.: +45-31863250

† These authors have contributed equally to this work and should be considered co-first authors.

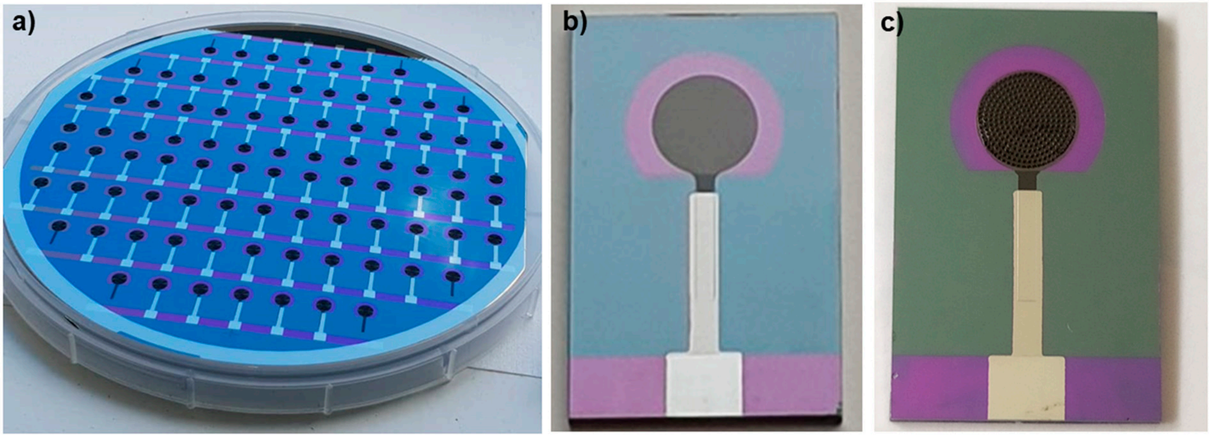

Figure S1. Photographs of (**a**) 6 inch wafer containing the 3D carbon micropillar electrodes; (**b**) 2D single chip and (**c**) 3D single chip with 12.6 mm^2 footprint area containing the 284 pyrolytic carbon micropillars.

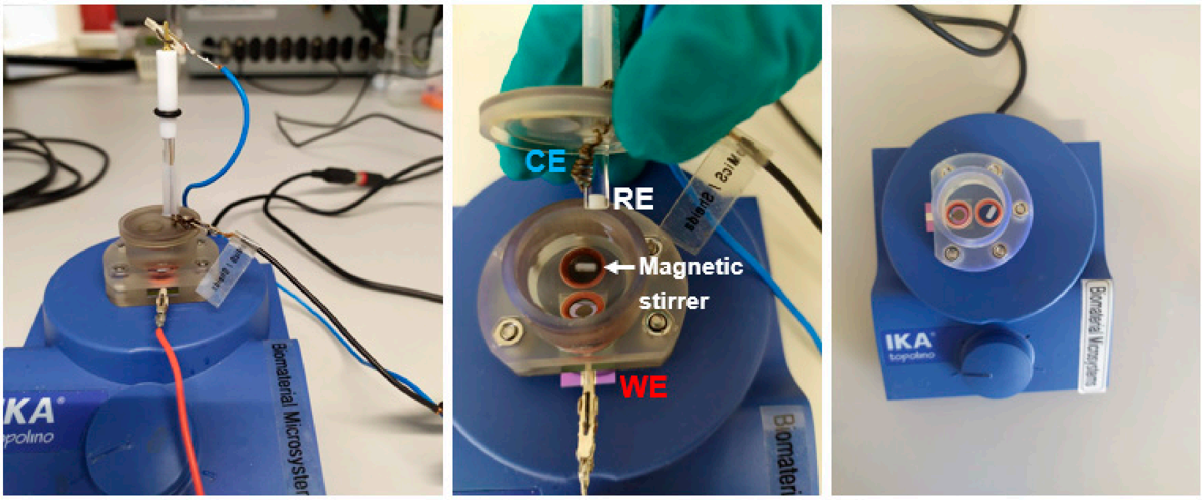

Figure S2. Top and side view photographs of the custom-made electrochemical cell with three electrodes arrangement. RE: reference electrode; CE: counter electrode; WE: working electrode.

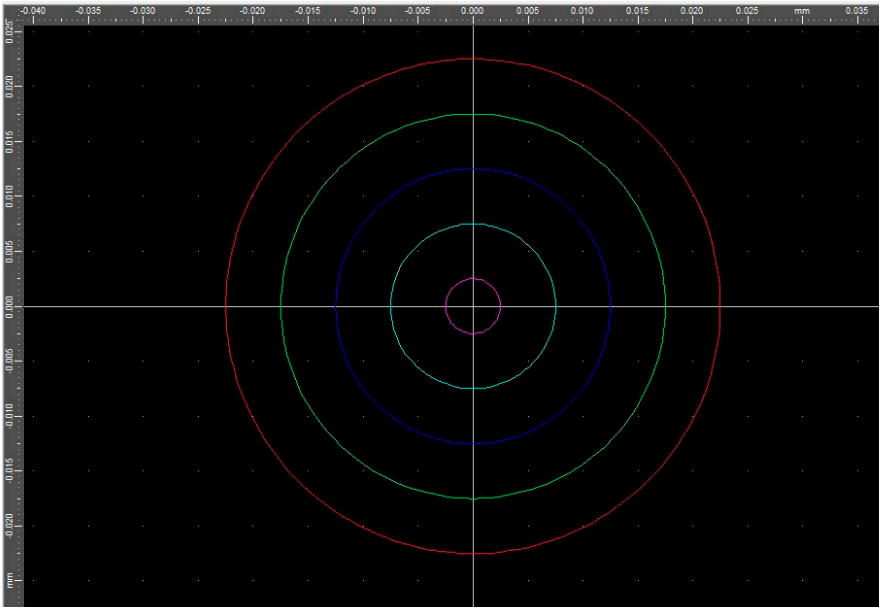

Figure S3. Schematic illustration of the laser pattern utilized for removing the PDA coating layer from the tip of a micropillar.
